# Epidemiology of low birth weight in Iran: A systematic review and meta-analysis

**DOI:** 10.1016/j.heliyon.2020.e03787

**Published:** 2020-05-22

**Authors:** Mehdi Shokri, Parviz Karimi, Hadis Zamanifar, Fatemeh Kazemi, Milad Azami, Gholamreza Badfar

**Affiliations:** a Department of Pediatrics, School of Medicine, Ilam University of Medical Sciences, Ilam, Iran; b School of Nursing and Midwifery, Ahvaz Jundishapour University of Medical Sciences, Ahvaz, Iran; c Student Research Committee, Qazvin University of Medical Sciences, Qazvin, Iran; d School of Medicine, Ilam University of Medical Sciences, Ilam, Iran; e Department of Pediatric, Faculty of Medicine, Ahvaz Jundishapour University of Medical Sciences, Ahvaz, Iran

**Keywords:** Public health, Pediatrics, Prevalence, Iran, Risk factors, Low birth weight, meta-analysis

## Abstract

**Introduction:**

Low birth weight (LBW) is an important general health indicator. The present study was conducted to evaluate the prevalence and risk factors of LBW in Iran.

**Method:**

This meta-analysis was reported based on the PRISMA guidelines. All stages were independently performed by two authors. This review is registered with PROSPERO (CRD42020163446). We searched epidemiological studies at international databases of Scopus, Embase, Science Direct, PubMed/Medline, CINAHL, EBSCO, Cochrane Library, Web of Science, and Google Scholar search engine, as well as Iranian databases of SID, IranDoc, Iranian National Library, Barakat Knowledge Network System, RICST and Magiran using MeSH keywords without time limit until 2019. After selecting the studies, applying the inclusion and exclusion criteria, data extraction and qualitative assessment, the data were analyzed based on random effects model using Comprehensive Meta-Analysis Software version 2. P < 0.05 was considered significant.

**Results:**

The prevalence of LBW in Iran was 7.95% (95% confidence interval [CI]: 7.36–8.58) in 62 studies with a sample size of 301,839 newborns. The prevalence of LBW in girls and boys was 8.41% (95%CI: 7.47–9.45) and 6.67% (95%CI: 5.86–7.59), respectively. The girls-to-boys odds ratio of LBW was 1.25 (95%CI: 1.13–1.39, P < 0.001) very LBW and extremely LBW prevalence was estimated to be 0.61% (95%CI: 0.40–0.93) and 0.29% (95% CI: 0.18–0.45), respectively. The risk factors for LBW were age of >35 versus [vs.] ≤35 (P = 0.024), age of <18 vs. ≥18 (P < 0.001), education of middle school and lower vs. high school and higher (P < 0.001), weight under 50 kg (P = 0.001), employed vs. housekeeper (P < 0.001), inadequate prenatal care (P = 0.046), interval with previous pregnancy <2 vs. >2 (P < 0.001), prematurity (P < 0.001), history of LBW (P < 0.001), multiple birth (P < 0.001), abortion (P < 0.001), vaginal bleeding (P < 0.001), hypertension (P = 0.001) and preeclampsia (P < 0.001).

**Conclusion:**

The results of this meta-analysis showed that LBW is prevalent in Iran. This study can be a national database for LBW that would be of interest to Iranian health policy-makers and planners.

## 1 Introduction

Low birth weight (LBW) is an important general health indicator, which is defined by the World Health Organization (WHO) as weight at birth less than 2500 g [1]. It is estimated that around 15.5% of newborns are born with LBW each year, and more than 95.6% of them are born in developing countries, while about 72% of LBW newborns are born in Asia and 8% are born in the eastern Mediterranean region, including Iran [2, 3]. The prevalence of LBW in developed and developing countries was estimated to be 5–7% and 19%, respectively [2].

LBW is one of the main causes of neonatal mortality, accounting for about 40% of all mortality among children under five years of age, and the mortality rate in LBW infants is approximately twenty times higher than heavier infants [1, 4]. The etiology of LBW is complex and is influenced by several factors such as demographic factors, maternal malnutrition, reproduction and socioeconomic factors such as inadequate care and difficult physical labor during pregnancy, family's deprivation of social protection, low levels of education and financial poverty [5, 6, 7]. Additionally, infections, multiple pregnancies and complications of pregnancy such as preeclampsia, maternal emotional distress, substance abuse, smoking, infertility, preterm labor, and intrauterine growth restriction (IUGR) are associated with LBW [8, 9, 10, 11].

LBW imposes an economic burden on the health care system, which is equal to one-third of the world's medical expenses [12]. In addition to health-related issues such as the need for hospital care, infants with LBW are at risk for chronic diseases and mental disabilities compared to infants with normal weight [13, 14]. LBW can be one of the major factors affecting growth disorder, cognitive development defects, and increased rate of diseases such as infectious diseases during pregnancy and childhood [15]. It is worth noting that recent epidemiological studies have shown that in people with LBW, the risk of developing chronic diseases in adulthood such as hypertension, coronary disease, kidney disease, diabetes, stroke and obesity is higher [16, 17]. Education level, age, poor diet, gravidity and parity, lack of proper prenatal care, as well as economic and social status are most important factors predicting of LBW risk [17, 18, 19, 20].

Several studies have been conducted in Iran on LBW [18, 19, 20, 21, 22, 23, 24]. In systematic reviews and meta-analyses, a complete picture of the dimensions of a problem in society can be presented by examining all relevant documentation and providing a general assessment [25, 26, 27]. Obviously, with the increase in the number of studies involved in the process of analysis, the confidence interval is reduced and the overall estimate is more reliable [27, 28]. Therefore, the present meta-analysis was conducted to determine the prevalence and risk factors of LBW in Iran.

## 2 Materials and methods

### 2.1 Study protocol

This meta-analysis was reported based on the Preferred Reporting Items for Systematic Reviews and Meta-Analyses (PRISMA) guidelines for systematic reviews and meta-analyses [26]. The study stages included the search strategy, the selection of studies, the qualitative assessment of studies, data extraction and statistical analysis. All these steps were independently performed by two authors. In the case of dispute, a third author was consulted. This review is registered with PROSPERO (CRD42020163446). Available from: https://www.crd.york.ac.uk/prospero/display_record.php?ID=CRD42020163446.

### 2.2 Search strategy

We searched epidemiological studies at nine international databases of Scopus, Embase, Science Direct, PubMed/Medline, CINAHL, EBSCO, Cochrane Library, Web of Science, and Google Scholar search engine, as well as six Iranian databases of Scientific Information Database (SID) (http://www.sid.ir/), Iranian Research Institute for Information Science and Technology (IranDoc) (https://irandoc.ac.ir), Iranian National Library (http://www.nlai.ir/), Barakat Knowledge Network System (http://health.barakatkns.com), and Regional Information Center for Science and Technology (RICST) (http://en.ricest.ac.ir/), and Magiran (http://www.magiran.com/) using MeSH and non MeSH keywords including “prevalence”, “Incidence”, “epidemiology”, “frequency”, “newborn”, “Infant”, “neonate”, “underweight”, “abnormal birth weight”, “birth outcome”, “low birth weight”, “preterm birth” and “Iran” without time limit until 2019. To perform a combined search, the "AND" and "OR" functions were used. An example of the PubMed search strategy were (prevalence OR epidemiology OR frequency) AND (newborn OR Infant OR neonate OR underweight OR abnormal birth weight OR birth outcome OR low birth weight OR and preterm birth) AND (Iran). The manual search was also done using the list of references in the selected or review articles.

### 2.3 Selection of studies

First, all related articles, whose affiliation included Iranian authors, were collected and after completing the search and removal of duplicates, two independent researchers screened the titles. After the screening process, we reviewed the summary. If there were doubts about eligibility of the article based on the abstract, the full text was examined and if the full text was not available, we contacted the author.

### 2.4 Inclusion and exclusion criteria

The inclusion criteria according to PICO (based on Evidence Based Medicine) [28] were: 1) **P**opulation: Epidemiologic studies (cross-sectional, cohort, and case-control) that examined the prevalence and risk factors of LBW; 2) **I**ntervention: Weight less than 2500 g to confirm LBW and subcategories include very low birth weight (VLBW), which is less than 1500 g, and extremely low birth weight (ELBW), which is less than 1000 g; 3) **C**omparison: Evaluation of the demographic, medical diseases, obstetrics and gynecology variable in infants with LBW and without LBW for risk factors; 4) **O**utcome: Estimating the prevalence and risk factors of LBW.

The exclusion criteria were: 1) Non-Iranian studies; 2) Studies with non-random sample size to estimate the prevalence of LBW; 3) Non-related studies; 4) Duplicate studies; 5) Case reports, Case series, Letter to Editor, Editorial, Commentary and review; and 6) Low quality studies.

### 2.5 Definition

LBW defined by the WHO as weight at birth less than 2500 g. Subcategories include VLBW, which is less than 1500 g, and ELBW, which is less than 1000 g [1].

### 2.6 Qualitative assessment

To assess the quality of selected studies, the Modified Newcastle-Ottawa scale for non-randomized studies and its adapted form for cross-sectional studies was used [29]. This checklist includes 7 questions, each receiving up to 10 stars. Therefore, the quality of studies was divided into three categories: unsatisfactory (less than 5 stars), satisfactory (5–7 stars) and good (8–10 stars). Finally, the scores given to the articles were compared by the two researchers and discussions were held on differences. The minimum score for entering the meta-analysis process was 5.

### 2.7 Data collection

The two authors independently extracted the data, including the first author, year of publication, year of study, sample size (total, girl, and boy), study design, LBW prevalence, VLBW prevalence, ELBW prevalence, geographic area of study, number of LBW and normal LBW in available variables. Any disagreement was resolved in consultation with a third person as a judge.

### 2.8 Evidence assessment

The overall methodological quality of each analysis was classified according to the Grading of Recommendations, Assessment, Development, and. Evaluation (GRADE), taking into account study limitations (risk of bias), inconsistency, imprecision, indirectness, and publication bias. Then, the quality of the evidence was divided into three categories: high, moderate, low or very low [30].

### 2.9 Statistical analysis

To determine the prevalence of LBW, the total number and the number of events were used. To estimate the risk factors of LBW, we used the total number and the number of events in both case and control groups and we calculated the odds ratio (OR) and 95% confidence interval (CI). Heterogeneity of studies was evaluated using Cochran's Q test and I^2^ index. In this regard, the interpretation is as follows: 0–24% may not be important, 25–49% may indicate a moderate heterogeneity, 50–75% indicates substantial heterogeneity, and over 75% indicates significant heterogeneity [31]. Moreover, in order to find the cause of heterogeneity, subgroup analysis and meta-regression were performed [32]. Based on the Dersimmonian-Laird test, the random effects model was used in this study to combine the data [33]. To ensure the strength and validity of the findings, sensitivity analysis was performed by omitting a study at a time [34]. Specifically, the subgroup analysis was performed based on year of study, type of study, sample size, study quality, geographical region and province. Any probable bias in the publication was evaluated using the Egger and Begg's tests [35]. Data were analyzed using Comprehensive Meta-Analysis Software (CMA) version 2. In this study, p < 0.05 was considered statistically significant.

## 3 Results

### 3.1 Overview of search

**Figure 1:**
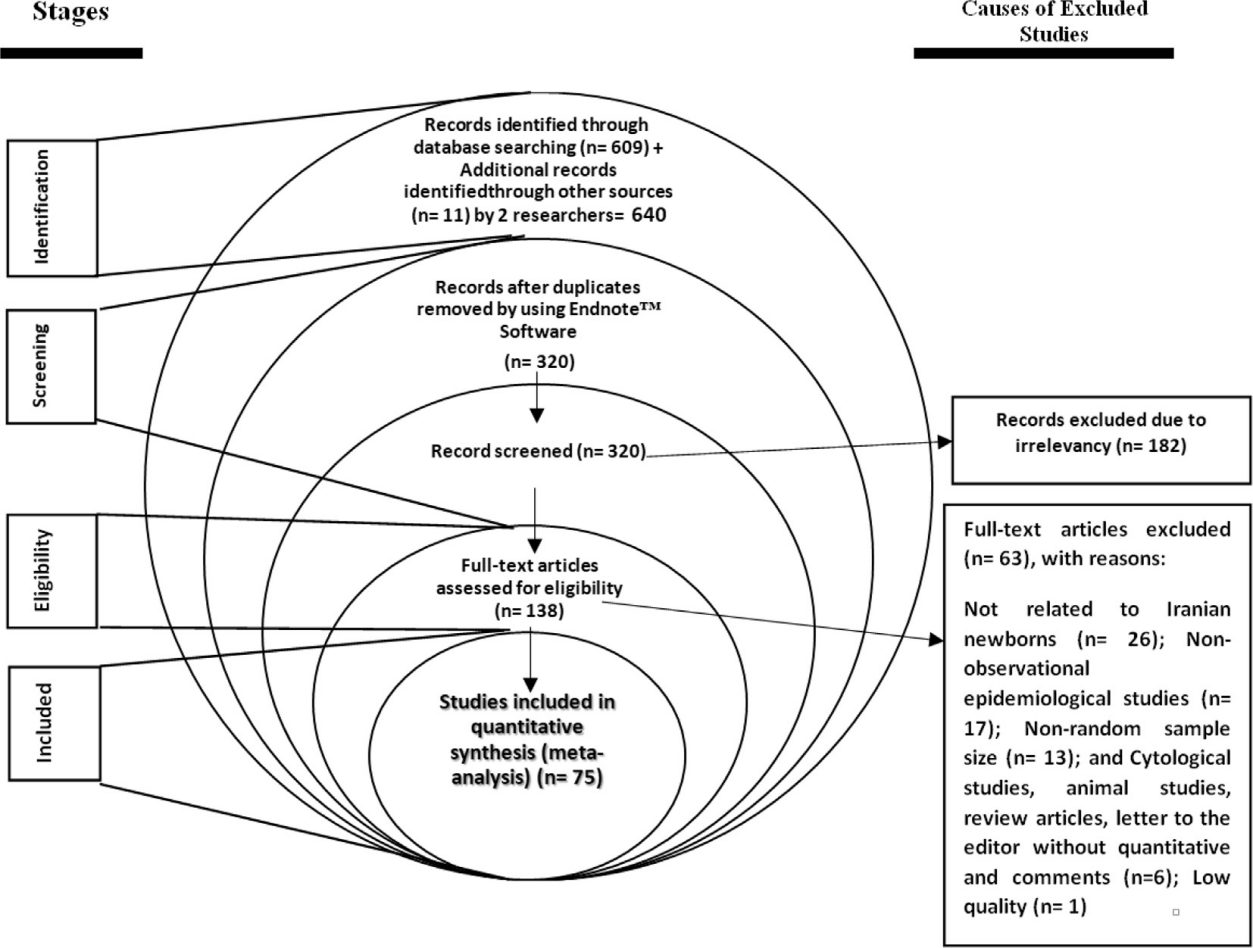
PRISMA flowchart.

In the initial search, 640 studies were obtained and 320 duplicate studies were deleted. After reviewing the full text of 138 related articles, 63 articles were excluded due to lack of necessary criteria and finally, 75 eligible studies entered the qualitative assessment stage (Figure 1). Table 1 shows the characteristics of each study.

**Table 1:** Summary of studies entered into meta-analysis.

Ref.	First author, Published Year	Year	Design	Place	Sample size (N∗)			LBW (%)	Quality
					All	Boy	Girl		
[18]	Mirahmadizadeh A, 2017	2014	Cross-sectional	Fars	3594	1811	1778	8.7	High
[19]	Momeni M, 2017	2014–5	Cross-sectional	Kerman	60273	29961	28226	9.42	High
[20]	Golestan M, 2011	2008	Cross-sectional	Yazd	5897			8.79	Moderate
[21]	Rafiei M, 2007	2005	Cross-sectional	Arak	4022	2051	1971	9.1	High
[22]	Delaram M, 2008	2005	Cross-sectional	Shahr-e-Kord	5102	2637	2465	8.5	Moderate
[23]	Shadzi Sh, 2000	1996–7	Cross-sectional	Isfahan	848	391	445	6	Moderate
[24]	Zarbakhsh Bhari M, 2012	2008–9	Cross-sectional	Giulan	32471			6.95	High
[36]	Hajian K, 2000	1998	Cross-sectional	Babol	1087	550	528	6.2	Moderate
[37]	Eslami Z, 2002	1999	Cross-sectional	Yazd	5053	2612	2441	7.975	Moderate
[38]	Hosseini SZ, 2005	2002	Cross-sectional	Tonekabon	2016	1653	363	4.2	Moderate
[39]	Ershadi A, 2000	1997	Cross-sectional	Kashan	5505	2793	2712	586	Moderate
[40]	Bayat H, 2004	2000	Cross-sectional	Qazvin	250			9.1	High
[41]	Mirzarahimi M, 2009	2006	Cross-sectional	Ardabil	7353			6.4	High
[42]	Roudbari M, 2007	2004	Cross-sectional	Zahedan	1240	587	522	11.79	High
[43]	Karimian S, 2003	2000	Cross-sectional	Qom	1927	985	921	11.79	Moderate
[44]	Davoudi N, 2012	2010	Cross-sectional	Mashhad	2674			11.1	High
[45]	Mirzarahimi M, 2013	2010–11	Cross-sectional	Ardabil	6832			6.32	Moderate
[46]	Pasdar Y, 2012	2010	Cross-sectional	Kermanshah	32450			5.7	Moderate
[47]	Hashemian Nejad N, 2014	2011–12	Cross-sectional	Sabzevar	7599			6.32	High
[48]	Karamzad N, 2016	2014	Case-control	Tabriz					Moderate
[49]	Chaman R, 2013	2011	Cross-sectional	Yasuj	1000			7.19	High
[50]	Khoori E, 1999	1996	Cross-sectional	Gorgan	2183	1107	1076	6.3	Moderate
[51]	Wafaie SM, 2005	2004	Case-control	Neishabour					Moderate
[52]	Zahed Pasha Y, 2004	2000	Cross-sectional	Babol	2228	1134	1082	7.7	High
[53]	Yousefi J, 2015	2007–8	Cross-sectional	Mashhad	866			16.5	Moderate
[54]	Khorshidi M, 2013	2011–12	Cross-sectional	Mazandaran	3792	1899	1893	2.9	Moderate
[55]	Rafati S, 2005	2002–3	Case-control	Tehran					High
[56]	Talebian MH, 2013	2009	Cross-sectional	Isfahan	9579			9.5	High
[57]	Jafari F, 2010	2004	Cross-sectional	Zanjan	4510	2368	2142	6.80	Moderate
[58]	Vahdaninia M, 2008	2005	Cross-sectional	Tehran	3734				High
[59]	Taheri FA, 2006	2004	Cross-sectional	Birjand	2558			7.9	Moderate
[60]	Tootoonchi P, 2007	2005–6	Cross-sectional	Tehran	905	395	514	8.6	High
[61]	YounesiI F, 2008	2004–7	Cross-sectional	Fars					Moderate
[62]	Saeedi R, 2013	2012	Case-control	Mashhad					High
[63]	Ranjbaran M, 2015	2013–4	Cross-sectional	Arak	461	221	240	6.72	High
[64]	Nachvak SM, 2012	2002–7	Cross-sectional	Tabriz					Moderate
[65]	Ahmadi P, 2017	2005–9	Cross-sectional	Tehran	600	237	312	9.5	High
[66]	Safari M, 2016	2013	Cross-sectional	Garmsar	681	340	340	4.7	High
[67]	Mahmoodi Z, 2013	2012	Case-control	Tehran					High
[68]	Mosayebi Z, 2004	1996	Cross-sectional	Tehran	10187			7.04	Moderate
[69]	Tabande A, 2007	2003–4	Cross-sectional	Gorgan	350			8.57	High
[70]	Mousa-farkhani E, 2002	2001	Cross-sectional	Quchan	803	426	377	12	Moderate
[71]	Shakiba M, 2008	2000–1	Cross-sectional	Rasht	5987			4.92	High
[72]	Veghari G, 2009	2007	Cross-sectional	Gorgan	2881			9.8	Moderate
[73]	Nili F, 2002	1999–2000	Cross-sectional	Tehran	2357			16	Moderate
[74]	Fadaei B, 2009	2009–10	Case-control	Isfahan					High
[75]	Fallah MH, 2008	2007	Cross-sectional	Yazd	941			9.35	Moderate
[76]	Eshraghian M, 2008	1995–6	Case-control	Tehran					High
[77]	Eghbalian F, 2007	2004–5	Cross-sectional	Hamedan	1500	812	688	19.1	Moderate
[78]	Tayebi T, 2013	2010	Cohort	Sari					High
[79, 80]	Bahrami N, 2014	2010	Cross-sectional	Qazvin	3076	1572	1407	6.7	High
[81]	Shahri P, 2012	2008	Cross-sectional	Ahvaz	808	379	429	4.9	High
[82]	Tabatabi S, 2010	2007	Cross-sectional	Tehran	2050			7.7	High
[83]	Eftaekhar H, 2007	2005	Case-control	Bandar Abbas					High
[84]	Koohdani F, 2010	2001–6	Case-control	Tehran					Moderate
[85]	Garmaroudi Gh, 2001	1996–7	Cross-sectional	Tehran	5893			4.395	Moderate
[86]	Sharifirad G, 2012	2010	Cross-sectional	Isfahan	225			7.10	Moderate
[87]	Faramarzi M, 2005	2001–3	Cross-sectional	Babol	3275			11.20	High
[88]	Nojomi M, 2006	2003	Cross-sectional	Tehran	430			12.79	High
[89]	Sobhi A, 2013	2008–2011	Cross-sectional	Fariman	7763			6.1	Moderate
[90]	Khojasteh F, 2016	2014	Cross-sectional	Zahedan	2227			4.84	Moderate
[91]	Delvarianzadeh M, 2007	2005	Cohort	Shahrood	424			13	Moderate
[92]	Sharifzadeh F, 2012	2008	Cohort	Tehran	576			13.02	High
[93]	Moghadam-Banaem L, 2010	2008	Cross-sectional	Tehran	344			3.5	High
[94]	Goujani R, 2014	2011	Cross-sectional	Rafsanjan	5532	2827	2685	7.066	High
[95]	Hosseini M, 2009	2004–5	Cohort	Tehran-Shemiran	610			11.79	Moderate
[96]	Alizadeh Sh, 2014	2010–11	Cross-sectional	Guilan	590			4.10	High
[97, 98]	Omani-Samani R, 2018	2015	Cross-sectional	Tehran	4859			5.16	High
[99]	Rafiei M, 2008	2004	Cross-sectional	Arak	10241	5241	5000	8.99	High
[100]	Judipour Z, 2015	2013	Cross-sectional	Sistan and Baluchestan	1712			9.3	Moderate
[101]	Amani R, 2000	1995–6	Cross-sectional	Ahvaz	876			7.3	Moderate
[102]	Oskouie F, 2006	2005	Cross-sectional	Tehran	1000			14.7	Moderate
[103]	Adlshoar M, 2005	2003	Cohort	Rasht	2500			5.2	Moderate
[104]	Golestan M, 2008	2004	Cohort	Yazd	6016			8.4	Moderate
[105]	Momenabadi V, 2017	2015	Cross-sectional	Shiraz	250	1811	1778	18	High

∗ Number.

### 3.2 LBW, VLBW, and ELBW prevalence

**Figure 2:**
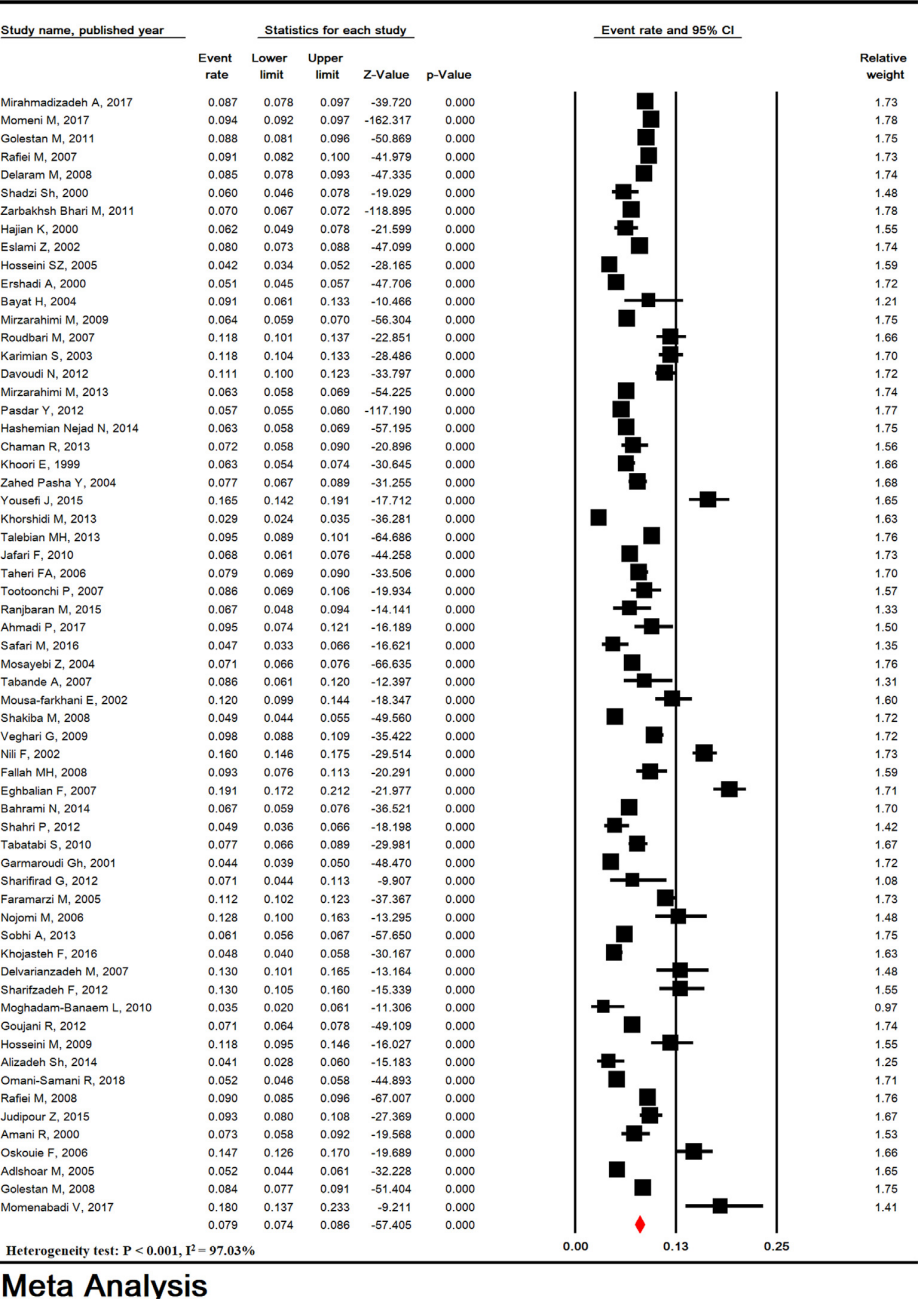
Prevalence of low birth weight in Iran.

**Figure 3:**
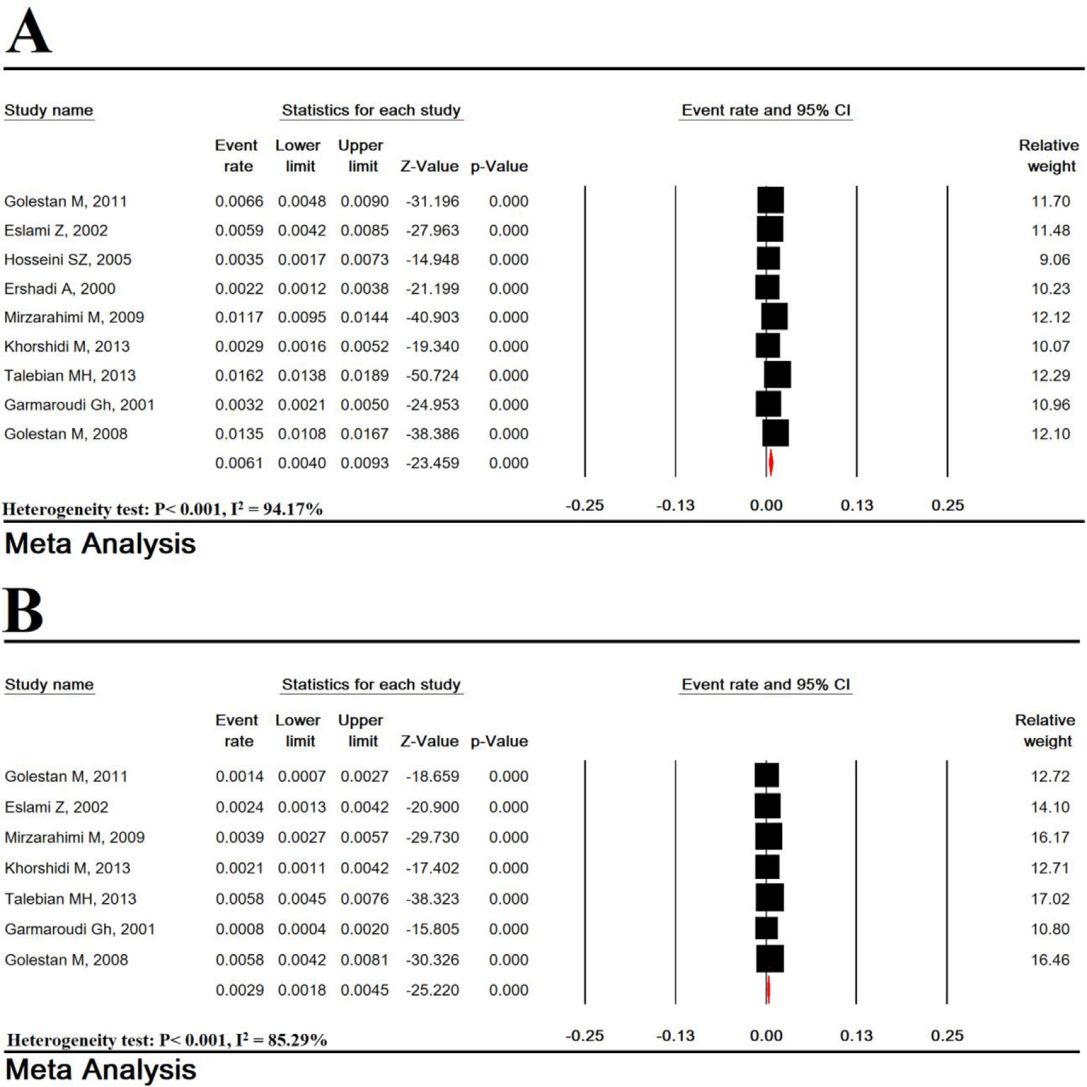
Prevalence of very low birth weight (A), and extremely low birth weight (B).

Total heterogeneity for prevalence of LBW, VLBW, and ELBW was very high in the studies (Heterogeneity test: P < 0.001, I^2^ = 97.03% for LBW, P < 0.001, I^2^ = 94.17% for VLBW, and P < 0.001, I^2^ = 85.29% for ELBW). The prevalence of LBW in Iran in 62 studies with sample size of 301,839 infants was estimated to be 7.95% (95% CI: 7.36–8.58). The lowest and highest LBW were related to studies in Mazandaran (2011-2) (2.9%) and Hamadan (2004–5) (19.1%), respectively (Figure 2). VLBW and ELBW prevalence was estimated to be 0.61% (95% CI: 0.40–0.93) and 0.29% (95% CI: 0.18–0.45), respectively (Figure 3).

### 3.3 Subgroup analysis

The subgroup analysis of LBW is shown in Table 2. The variables of geographical area (P = 0.066), study design (P = 0.196), quality (P = 0.957), sample size (P = 0.241) and year of studies (P = 0.088) were not significant, but the subgroup analysis of provinces (P < 0.001) was significant (Table 2).

**Table 2:** Subgroup analysis of LBW based on region, quality of studies, study design, Provinces, year, and sample size.

Variable		Studies (N^a^)	Sample (N)		Heterogeneity		95%CI^b^	Pooled prevalence (%)
			All	Event	I^2^	P-Value		
Region	Center	29	94154	7577	95.69	<0.001	7.37–9.19	8.23
	East	9	27442	2106	96.87	<0.001	7.05–11.32	8.95
	North	15	73969	4916	95.75	<0.001	5.57–7.53	6.48
	South	7	72333	6602	92.70	<0.001	7.02–9.81	8.31
	West	2	33950	2136	99.73	<0.001	3.04–31.20	10.66
	Test for subgroup differences: Q = 8.80, df(Q) = 4, P = 0.066							
Quality	High	30	173298	14254	95.45	<0.001	7.31–8.71	7.98
	Moderate	32	128541	9084	97.68	<0.001	6.94–9.08	7.95
	Test for subgroup differences: Q = 0.003, df(Q) = 1, P = 0.957							
Study design	Cross-sectional	57	291713	22500	98.17	<0.001	7.21–8.47	7.81
	Cohort	5	10126	837	94.14	<0.001	7.05–13.17	9.69
	Test for subgroup differences: Q = 1.67, df(Q) = 1, P = 0.196							
Provinces	Khuzestan	2	1684	104	75.86	0.042	4.08–8.89	6.05
	Qazvin	2	3326	229	51.47	0.151	55.6–9.68	7.36
	Kerman	2	65805	6069	96.99	<0.001	6.17–10.82	8.20
	Mazandaran	5	12398	800	98.02	<0.001	3.48–9.68	5.85
	Tehran	12	29811	2259	97.67	<0.001	6.65–11.54	8.79
	Yazd	4	17907	1515	8.14	0.352	80.5–8.91	8.47
	Sistan and Baluchestan	3	5179	413	96.41	<0.001	4.94–13.19	8.16
	Hamedan	1	1500	287	0	-	17.19–21.17	19.10
	Kermanshah	1	32450	1850	0	-	5.45–5.96	5.70
	Qom	1	1927	227	0	-	10.43–13.32	11.80
	Markazi	3	14724	1318	31.55	-	8.279.59	8.91
	Chaharmahal va Bakhtiari	1	5102	434	0	-	7.77–9.30	8.50
	Kohgiloyeh and Boyerahmad	1	1000	72	0	-	5.75–8.98	7.20
	Semnan	2	1105	87	95.63	<0.001	2.83–20.37	7.95
	South Khorasan	1	2558	202	0	-	6.92–9.01	7.90
	Ardabil	2	14185	902	0	0.845	5.97–6.78	6.36
	Fars	2	3844	358	95.60	<0.001	5.92–24.41	12.48
	Gilan	4	41548	2709	93.71	<0.001	4.18–6.84	5.36
	Golestan	3	5414	450	89.85	<0.001	5.81–11.14	8.09
	Isfahan	4	16157	1257	96.88	<0.001	4.43–10.16	6.75
	Razavi Khorasan	5	19705	1491	97.98	<0.001	6.77–13.72	9.70
	Zanjan	1	4510	307	0	-	6.10–7.57	6.80
	Test for subgroup differences: Q = 560.43, df(Q) = 21, P < 0.001							
Year	1996–2005	31	91883	7356	96.97	<0.001	7.49–9.71	8.54
	2006–2015	31	209956	15982	97.10	<0.001	6.69–8.18	7.40
	Test for subgroup differences: Q = 2.91, df(Q) = 1, P = 0.088							
Sample	≤1000	22	13838	1305	90.43	<0.001	7.48–10.58	8.91
	1001–10000	35	142379	10606	97.35	<0.001	6.68–8.39	7.49
	>10000	5	145622	11426	99.14	<0.001	6.09–9.23	7.51
	Test for subgroup differences: Q = 2.84, df(Q) = 2, P = 0.241							

a Number.

b Confidence interval.

### 3.4 LBW based on gender

**Figure 4:**
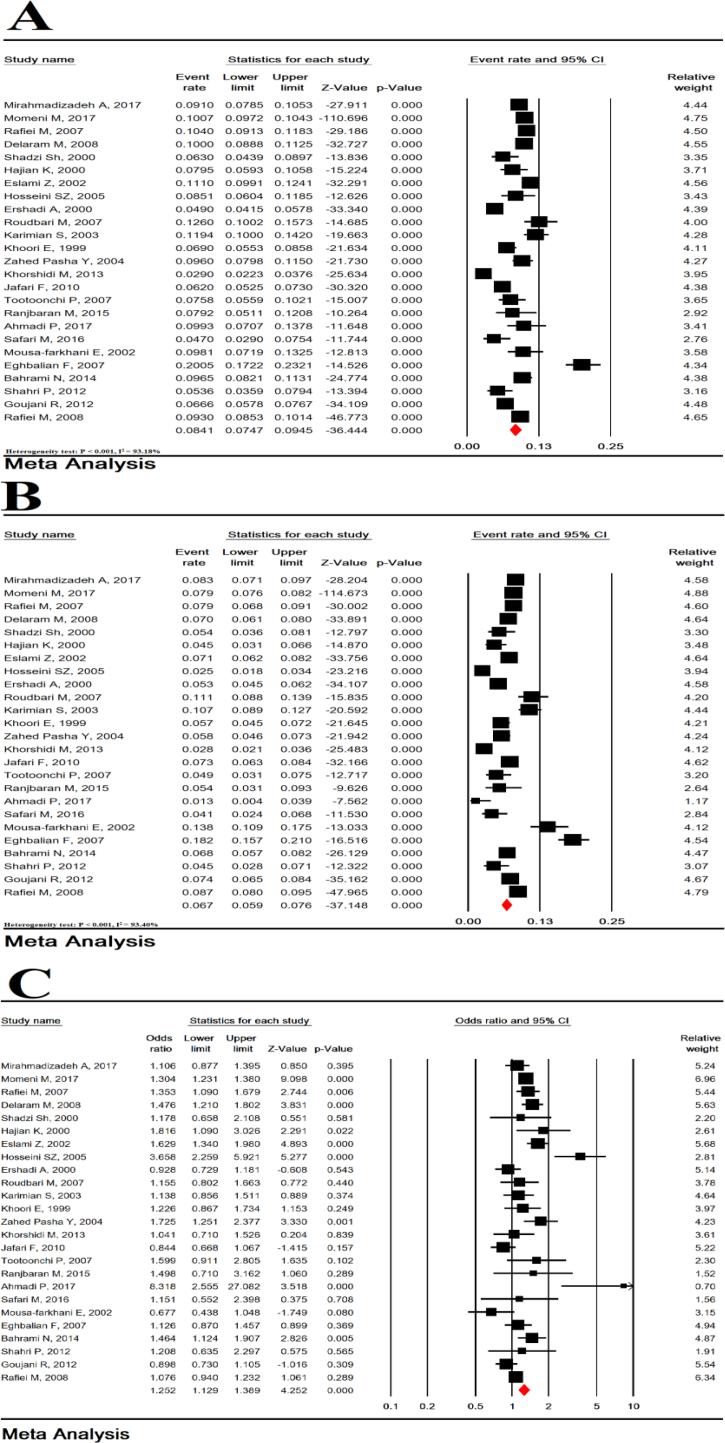
Prevalence of LBW in girls (A), boys (B) and an odds ratio of girls -to-boys (C).

Total heterogeneity was very high for prevalence of LBW in girls and boys (Heterogeneity test: P < 0.001, I^2^ = 93.18% for girls gender and heterogeneity test: P < 0.001, I^2^ = 93.40% for boys gender). The prevalence of LBW in girls in 25 studies with a sample size of 60,557 infants was 8.41% (95% CI: 7.47–9.45). The prevalence of LBW in boys in 25 studies with a sample of 64,989 infants was 6.67% (95% CI: 5.86–7.59) (Figure 4-A,B). The female-to-male OR of LBW was 1.25 (95% CI: 1.13–1.39, P < 0.001) (Figure 4-C).

### 3.5 LBW based on place of residence

**Figure 5:**
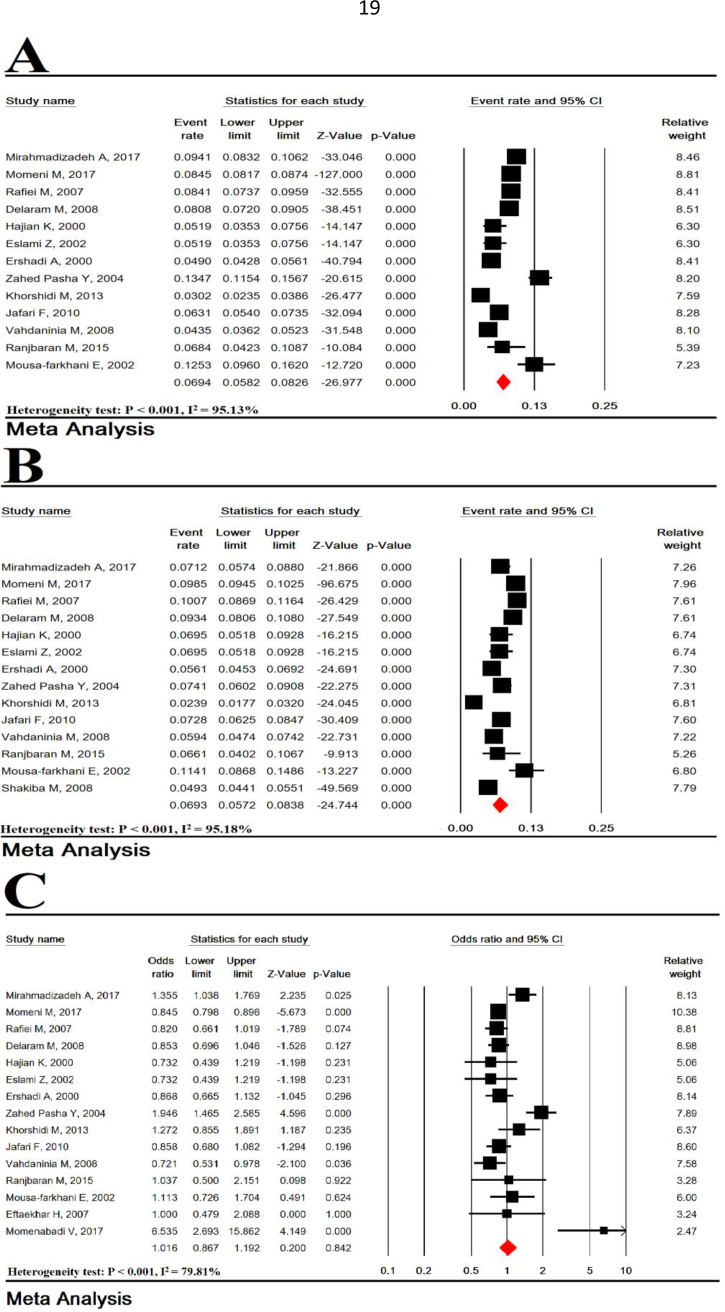
Prevalence of LBW in studies of urban (A), rural (B), and an odds ratio of urban -to- rural (C).

Total heterogeneity was very high for prevalence of LBW in urban and rural studies (Heterogeneity test: P < 0.001, I^2^ = 95.13% for urban studies and heterogeneity test: P < 0.001, I^2^ = 95.18% for rural studies). The prevalence of LBW in urban areas (14 studies with a sample size of 41,454 infants) and rural areas (13 studies with a sample size of 58,593 infants) were 6.94% (95% CI: 5.82–8.26) and 6.93% (95% CI: 5.72–8.38). The urban-to-rural OR of LBW was 1.01 (95% CI: 0.86–1.19; P = 0.842) (Figure 5).

### 3.6 Risk factors for LBW

The demographic risk factors for LBW, including age of >35 versus (vs.) ≤ 35 (1.41 [95% CI: 1.04–1.90], P = 0.024), age of <18 vs. ≥18 years (1.39 [95% CI: 1.20–1.61], P < 0.001), education of middle school and lower vs. high school and higher (1.56 [95% CI: 1.28–1.90], P < 0.001), weight under 50 kg (2.49 [95% CI: 1.45–4.26], P = 0.001), employed vs. housewife (2.40 [95% CI: 1.52–3.80], P < 0.001) were significant, but smoking (3.52 [95% CI: 0.85–14.48], P = 0.081) was not significant (Table 3).

**Table 3:** Rick factors of LBW.

Variable	Studies (N^c^)	Sample (N)				Heterogeneity		95%CI^d^	OR^e^	P-Value	Model	Bias test	
		Case		Control		I^2^	P-Value					Egger	Beggs
		All	Event	All	Event								
Age of >35 vs. ≤35	23	55534	4975	48959	4931	89.42	<0.001	1.04–1.90	1.41	0.024	Random	0.021	0.561
Age of <18 vs. ≥18^a^	27	7346	919	102024	9345	59.23	<0.001	1.20–1.61	1.39	<0.001	Random	0.859	0.587
Interval with previous pregnancy <2 vs. ≥2^b^	12	2482	498	5365	750	75.25	<0.001	1.46–3.14	2.14	<0.001	Random	0.658	0.631
Wight under 50 kg	5	994	228	4442	868	80.73	<0.001	1.45–4.26	2.49	0.001	Random	0.267	0.462
Unwanted pregnancy	10	543	3092	8948	837	90.30	<0.001	0.90–3.00	1.64	0.106	Random	0.125	0.210
Inadequacy prenatal care	10	216	1343	10371	1565	72.17	<0.001	1.00–2.30	1.54	0.046	Random	0.898	0.720
Education of middle school and lower vs. high school and higher	17	32539	3999	42266	3788	77.26	<0.001	1.28–1.90	1.56	<0.001	Random	0.281	0.621
Employed vs. housekeeper	17	6275	807	21363	2209	91.74	<0.001	1.52–3.80	2.40	<0.001	Random	0.526	0.433
Smoking	5	174	32	6473	776	80.05	<0.001	0.85–14.48	3.52	0.081	Random	0.504	0.462
Prematurity	20	9639	4626	52130	5270	99.49	<0.001	4.99–38.49	13.86	<0.001	Random	0.050	0.215
History of LBW	10	778	269	13425	1189	87.00	<0.001	1.91–6.67	3.57	<0.001	Random	0.154	0.591
Cesarean section	11	10289	1181	15991	1366	93.31	<0.001	0.76–1.62	1.11	0.584	Random	0.342	0.640
Multiple birth	13	3164	647	29991	2230	97.79	<0.001	4.82–36.12	13.20	<0.001	Random	0.031	0.200
Nulliparity	21	17357	1861	24815	2019	97.60	<0.001	0.99–1.32	1.14	0.059	Random	0.186	0.215
Abortion	8	1857	253	5700	872	93.10	<0.001	0.60–2.73	1.28	0.651	Random	0.448	0.901
Vaginal bleeding	7	522	219	4495	1095	34.52	0.165	1.83–3.57	2.56	<0.001	Random	0.392	0.548

a In many studies cut off for age is < 20 vs. ≥20.

b In many studies cut off for Interval with previous pregnancy <3 vs. ≥3.

c Number.

d Confidence interval.

e Odds ratio.

The obstetrics and gynecology risk factors for LBW, including inadequate prenatal care (1.54 [95% CI: 1.00–2.30], P = 0.046), interval with previous pregnancy <2 vs. > 2 years (2.14 [95% CI: 1.46–3.14], P < 0.001), prematurity (13.86 [95% CI: 4.99–38.49], P < 0.001), history of LBW (3.57 [95%CI: 1.91–6.67], P < 0.001), multiple birth (13.20 [95% CI: 4.82–36.12], P < 0.001), abortion (1.28 [95% CI: 0.60–2.73], P = 0.651), vaginal bleeding (2.56 [95% CI: 1.83–3.57], P < 0.001) but unwanted pregnancy (1.64 [95% CI: 0.90–3.00], P = 0.106), nulliparity (1.14 [95% CI: 0.99–1.32], P = 0.059) and cesarean section (1.11 [95% CI: 0.76–1.62, P = 0.584]) were not significant (Table 3).

**Figure 6:**
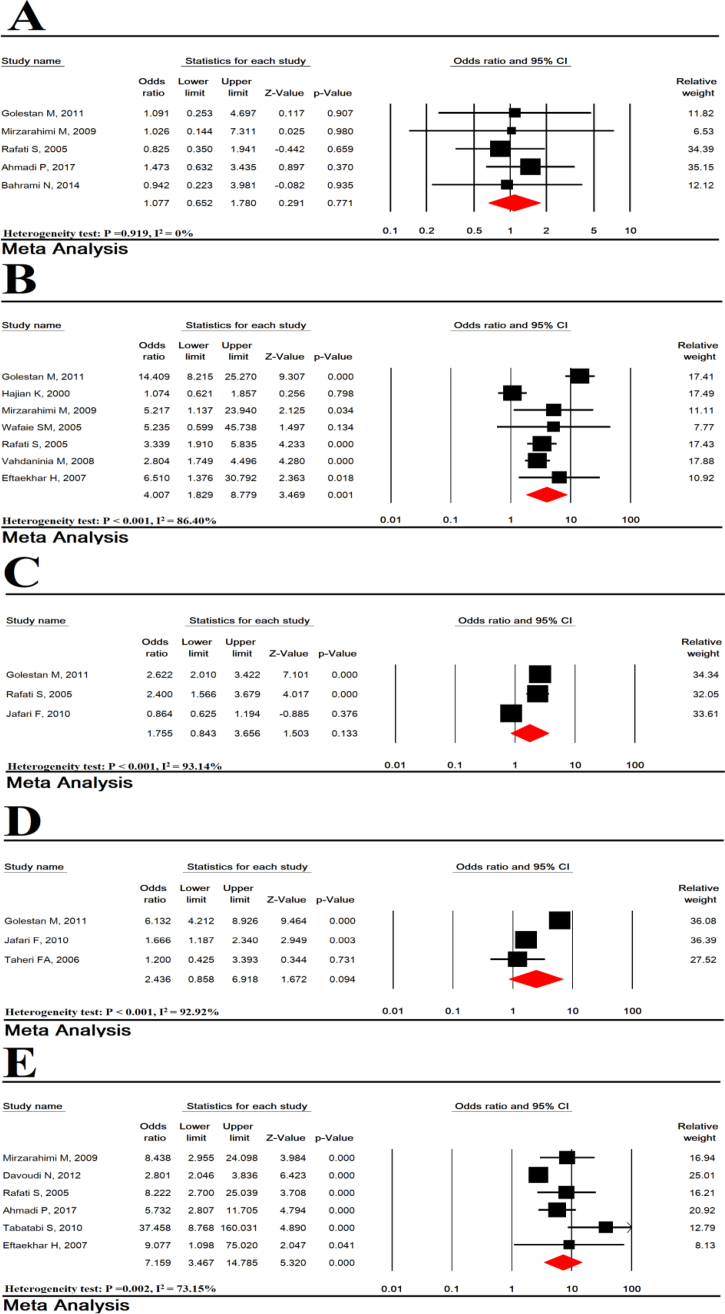
Relationship of low birth weight and diabetes mellitus (AND), hypertension (B), urinary tract infection (C), induced pregnancy hypertension (D) and preeclampsia (E).

Risk factors of medical diseases for LBW, including hypertension (P = 0.001) and preeclampsia (P < 0.001) were significant but diabetes mellitus (P = 0.77), urinary tract infection (P = 0.133), pregnancy-induced hypertension (0.094) were not significant (Figure 6).

### 3.7 Meta-regression

The meta-regression model showed that the changes in the prevalence of LBW were not significant based on the year of study (meta-regression coefficient: -0.003, 95% CI: -0.019 to 0.012, P = 0.663). In addition, this model was not significant for the prevalence of LBW in girls (meta-regression coefficient: -0.009, 95% CI: -0.035 to 0.17, P = 0.497) and boys (meta-regression coefficient: -0.003, 95% CI: -0.031 to 0.024, P = 0.801) and also VLBW (meta-regression coefficient: 0.067, 95% CI: -0.004 to 0.139, P = 0.065) and ELBW (meta-regression coefficient: 0.055, 95% CI: -0.041 to 0.151, P = 0.262) (SF [Supplementary figure] 1, 2).

### 3.8 Sensitivity analysis and publication bias

The sensitivity analysis showed a strong point estimate by eliminating a study at a time for prevalence of LBW, VLBW, and ELBW (SF 3, 4). Publication bias was not significant for the prevalence of LBW based on P-values of Egger and Begg's tests were 0.746 and 0.836, respectively. The publication bias was not significant for the female-to-male odds ratio of LBW based on Egger (P = 0.829) and Begg's test (P = 0.387). Publication bias is shown in SF 5 file in the form of a funnel plot.

## 4 Discussion

The Millennium Development Goals (MDGs) are aimed at reducing the mortality of children under the age of 5 to two-thirds. The most important factor that can affect the survival of infants is LBW. This is an important health index in any country [106]. One of the goals of sustainable development is to reduce the mortality rate of infants to below 12 per 1,000 live births in all countries by 2030. The neonatal mortality rates in Iran have been reported to be 13.3 infants according to the World Bank collection of development indicators [107], which could be due to improvements in Iran's national health system.

The prevalence of LBW in Iranian studies has been reported between 2.9% and 19.1% in different regions. In the present meta-analysis, the national prevalence of LBW in Iran among 301,839 infants was estimated to be 7.95%. The prevalence of LBW in 2015 was estimated to be in worldwide (14.6%), Sub-Saharan Africa (14.0%), Southern Asia (26.4%), Northern Africa (12.2%), Southeastern Asia and Oceania (12.2%), Central Asia (5.4%), Eastern Asia (5.3%), Western Asia (9.9%), Latin America and Caribbean (8.7%) [108]. Considering high heterogeneity of LBW prevalence in Iranian studies, subgroup analysis was done to find its cause, and province (P < 0.001) was the only significant factor. Therefore, the prevalence of LBW varies according to differences in health care quality, sample size, and socioeconomic and cultural conditions in different regions of Iran, so it should be considered by policy makers and health care providers.

The meta-regression model for LBW prevalence did not change significantly based on the year of study (between 1993 and 2017). In a systematic global review article, its prevalence was 17.5% in 2000 and 14.6% in 2015, and in West Asian countries, it was 9.9% in 2000 and 8.9% in 2015 [108].

The sickest and youngest infants are often missed from information systems, including those who die soon after birth, or are hospitalized elsewhere. The information system and communication system should be improved to obtain information about these vulnerable infants. Incorrect classification of premature infant mortality as “stillbirths” still exists. Since these infants are more likely to suffer from LBW, failure to consider mortality may lead to underestimation of the prevalence of LBW. Therefore, it is important that any newborn, whether alive or dead, is weighed at birth and that basic information including birth weight and gestational age is recorded in the information system [109]. Social and family demand for birth weight data is an issue that is not discussed. There is little information about family and community perceptions and the demand for birth weight measurement, including cultural barriers to birth weight measurement, especially in some areas of the community and for stillbirths.

Preterm delivery plays a major role in developing LBW. A systematic review and meta-analysis reported the prevalence of preterm labor to be 9.2% in Iran and considered it a relatively common problem in Iran [110].

In evaluating the effect of gender on LBW, we found that the prevalence of LBW in females was significantly higher than males. In a study conducted in Japan, there was a significant relationship between female gender and low birth weight [111], and it was also found that the mean birth weight of male infants was higher than that of female infants [112].

In the present study, demographic risk factors included maternal age, low education, being employed and low maternal weight. Regarding the age of the mother, young women (under the age of 19) and women of advanced maternal age (over 35) are more likely to suffer from LBW. This finding is similar to other studies [113, 114, 115]. The employment of pregnant women in hard, troublesome, and active jobs is among the factors affecting LBW, early delivery and fetal death [116]. Working conditions are also important predictors of the outcome of pregnancy and childbirth. Various studies have shown that type of occupation as well as working conditions may lead to LBW [117, 118, 119, 120]. Similarly, other studies have shown that economic status, education, and weight during pregnancy may play an important role [121].

In the present study, there was no significant relationship between smoking and LBW. But smoking should be considered as a dangerous side effect for pregnant women. Some studies show that any type of smoking during pregnancy may lead to LBW, cognitive impairment, respiratory problems, birth defects, early delivery, and even infant death [122, 123, 124, 125].

The risk factors for gynecologic and obstetric care in the present study included interval of less than 2 years with the previous pregnancy, inadequate prenatal care, prematurity, LBW history, multiple sclerosis, abortion and vaginal bleeding. In a review article in developing countries, maternal age of 35–49 years, illiteracy, inadequate antenatal care, delayed conception, and being in the poorest socioeconomic stratum were among the risk factors for increasing LBW [126].

Another review article emphasized the role of inter-pregnancy interval and found that it has significant effect on the short intervals between pregnancies for outcomes: extremely preterm birth [< 6 month adjusted odds ratio (aOR): 1.58 [1.40, 1.78], 6–11 month aOR: 1.23 [1.03, 1.46], moderate preterm birth (<6 m aOR: 1.41 [1.20, 1.65], 6–11 month aOR: 1.09 [1.01, 1.18]), low birthweight (<6 month aOR: 1.44 [1.30, 1.61], 6–11 month aOR: 1.12 [1.08, 1.17]), stillbirth (aOR: 1.35 [1.07, 1.71] and early neonatal death (aOR: 1.29 [1.02, 1.64]) [127]. A review article in Ethiopia showed that maternal age <20 years (aOR = 1.7; 95% CI: 1.5–2.0), BMI <18.5 kg/m2 (aOR = 5.6; 95% CI: 1.7–9.4), pregnancy interval <24 months (aOR = 2.8; 95%CI: 1.4–4.2), and prematurity (aOR = 6.4; 95% CI: 2.5–10.3) are among LBW risk factors [128].

In the present study, the relationship between caesarean section and LBW was not significant. Some studies show that LBW is higher in women with CS delivery. However, this conclusion is controversial, while in other studies, the risk of LBW was not reported to be higher in CS delivery [129].

The medical risk factors in the present study were LBW, hypertension and preeclampsia. The association between LBW and preeclampsia has been confirmed in other countries [130, 131, 132]. Other meta-analytical studies have shown the effect of anemia on LBW and Small for gestational age [133, 134]. It is recommended that attention be paid to thyroid disorders and LBW in future meta-analytical studies [135].

The strengths of this study: 1. We used a comprehensive search strategy to maximize the identification of all relevant literature, 2. Following the PRISMA protocol, we were able to provide the largest data on LBW in Iran to date, 3. We contacted the first author or the corresponding author to eliminate the ambiguity of the articles, 4. We used random effects model to integrate the data to provide a conservative estimate of the prevalence of LBW, and subgroup analysis and meta-regression model were performed to detect the cause of heterogeneity and to evaluate the publication bias. Limitations of the present study: 1. Search in national databases was limited due to limitations in combined search in these databases, 2. Studies on specific infants such as preterm infants, etc. or non-random sample sizes were excluded and the resulting estimate may be attributable to the general public, 3. In addition, there was a high heterogeneity between studies in the meta-analysis, and based on available data, we could attribute this difference to the provinces under study (P < 0.001), but there appears to be other causes, including differences in lifestyle, dietary habits, ethnicity (given that Iran has different ethnicities with different customs [136]) may also be effective, which could not be investigated using the available data.

## 5 Conclusion

The results of this meta-analysis showed that LBW is prevalent in Iran. Effective risk factors in LBW in Iranian population include low and high maternal age, low level of education, low maternal weight, maternal occupation, inadequate prenatal care, short interval with previous pregnancy, prematurity, LBW history, multiple sclerosis, abortion, vaginal bleeding, hypertension and preeclampsia, while many of these risk factors are manageable. Therefore, controlling these factors before and during pregnancy by health policymakers may lead to the birth of healthy infants and reduce the incidence of LBW.

## Declarations

### Author contribution statement

All authors listed have significantly contributed to the development and the writing of this article.

### Funding statement

This research did not receive any specific grant from funding agencies in the public, commercial, or not-for-profit sectors.

### Competing interest statement

The authors declare no conflict of interest.

### Additional information

No additional information is available for this paper

## Appendix A Supplementary data

Supplement file_V2 Supplement file_V2
