# Supplementary material for: Epidemiology of low birth weight in Iran: A systematic review and meta-analysis
Source: Heliyon. 2020 May 22;6(5):e03787. doi: 10.1016/j.heliyon.2020.e03787 (PMC7251772; doi:10.1016/j.heliyon.2020.e03787)
Supplement: Supplement file_V2 [file mmc1.docx]

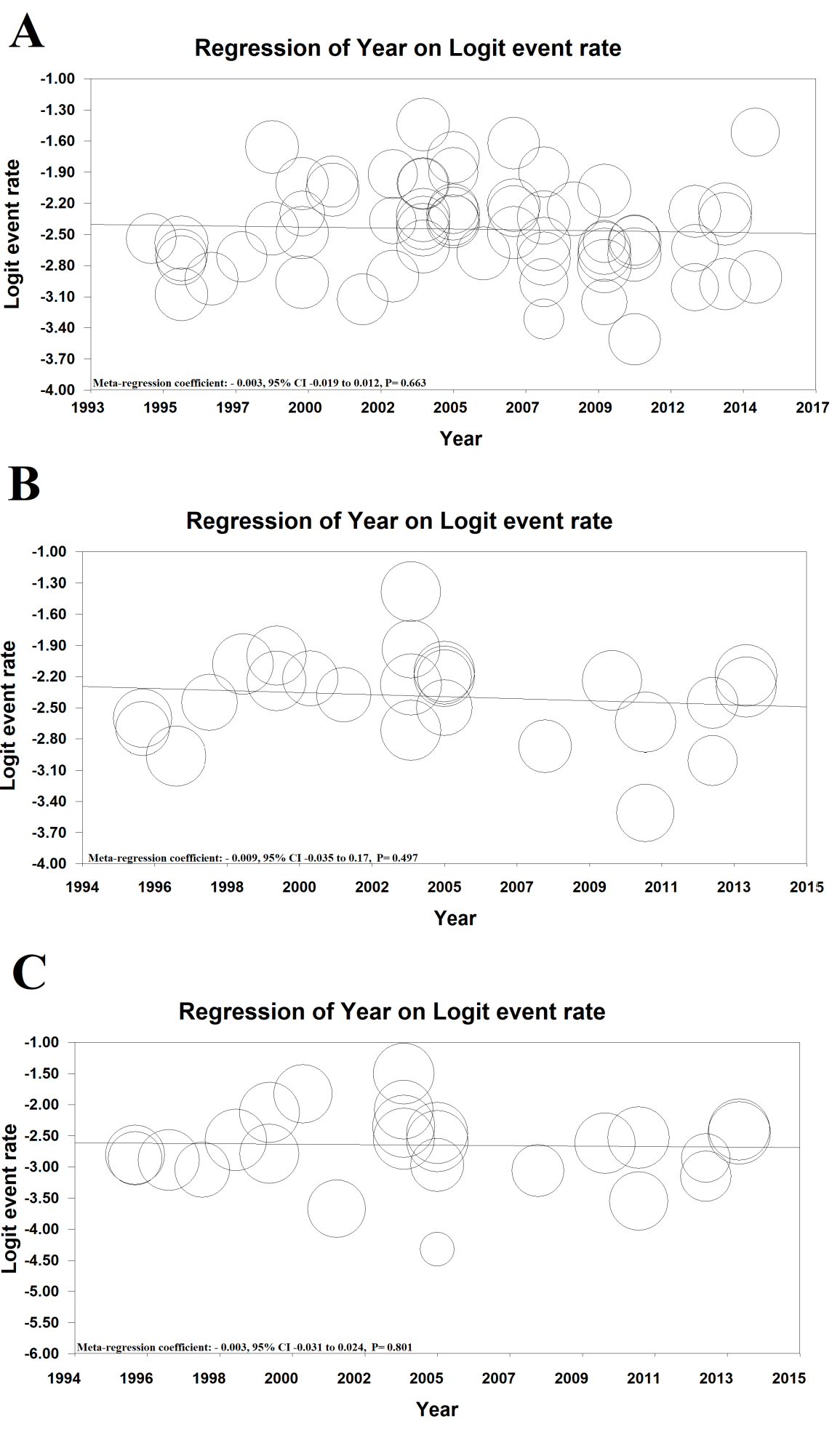
Figure 1: Regression of Logit event rate of LBW prevalence in total (A), girls (B) and boys (C) based on year of the studies


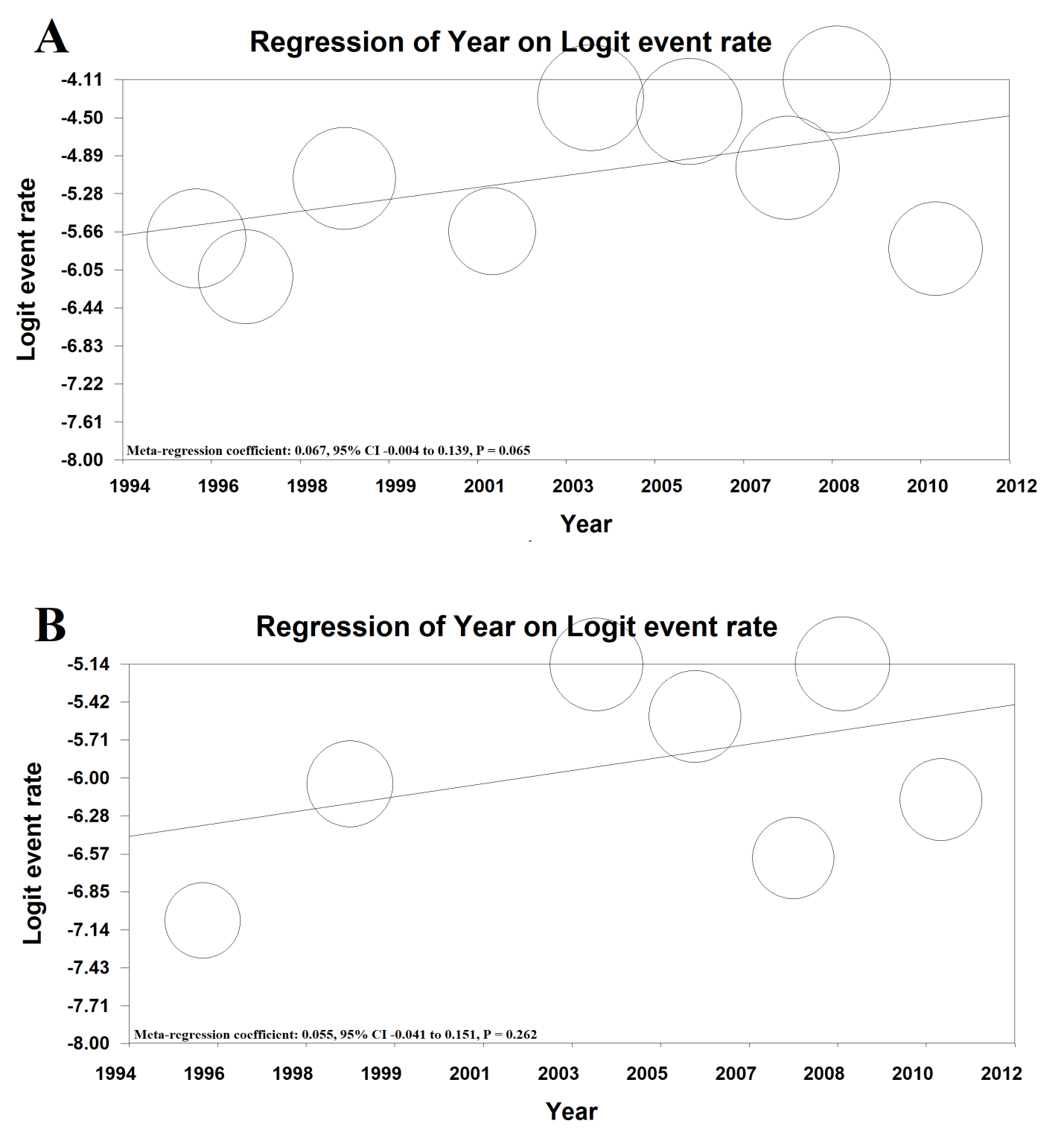
Figure 2: Regression of Logit event rate of prevalence of VLBW (A), and ELBW (B) based on year of the studies

Figure 3: Sensitivity analysis for prevalence of LBW in Iran
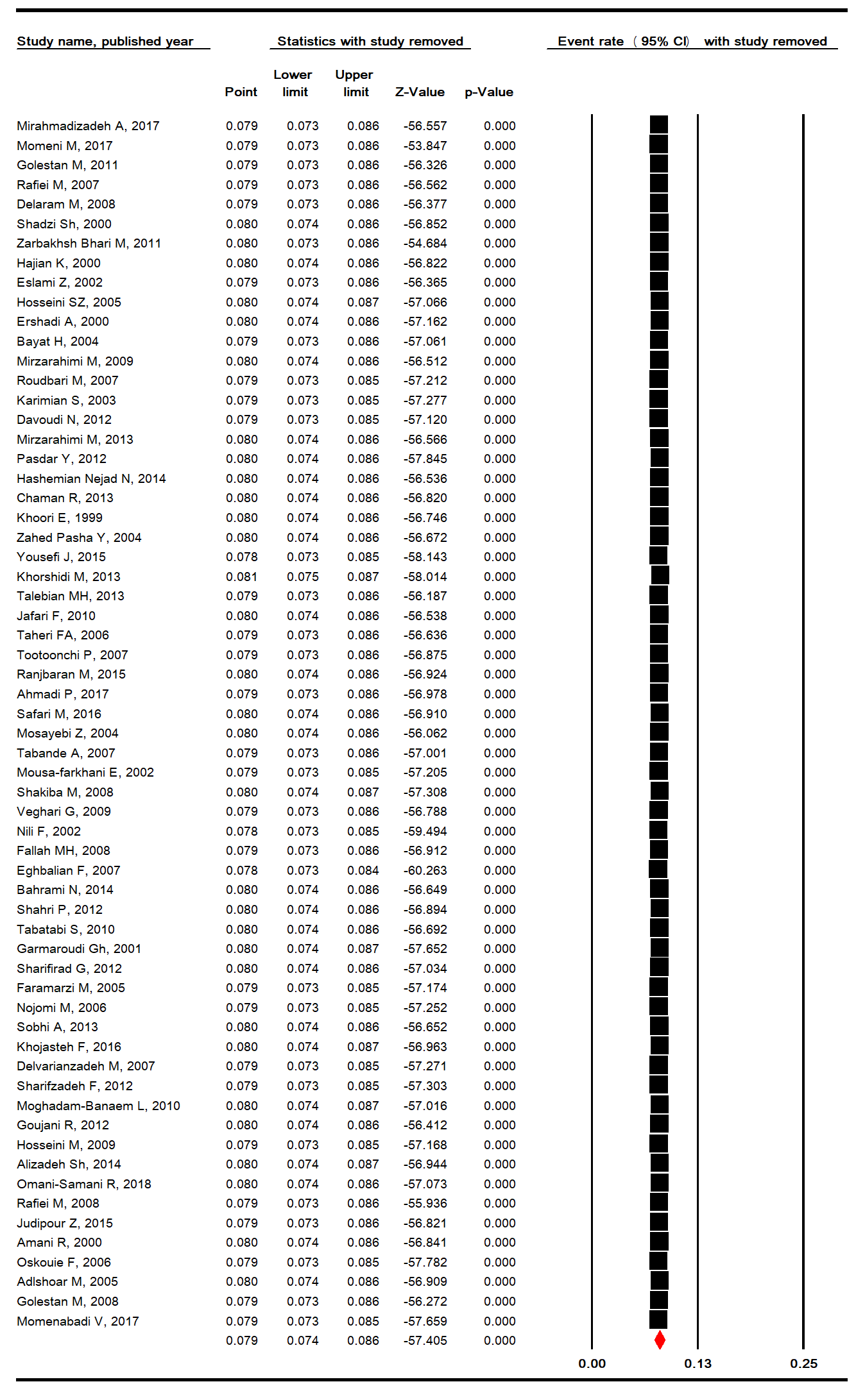


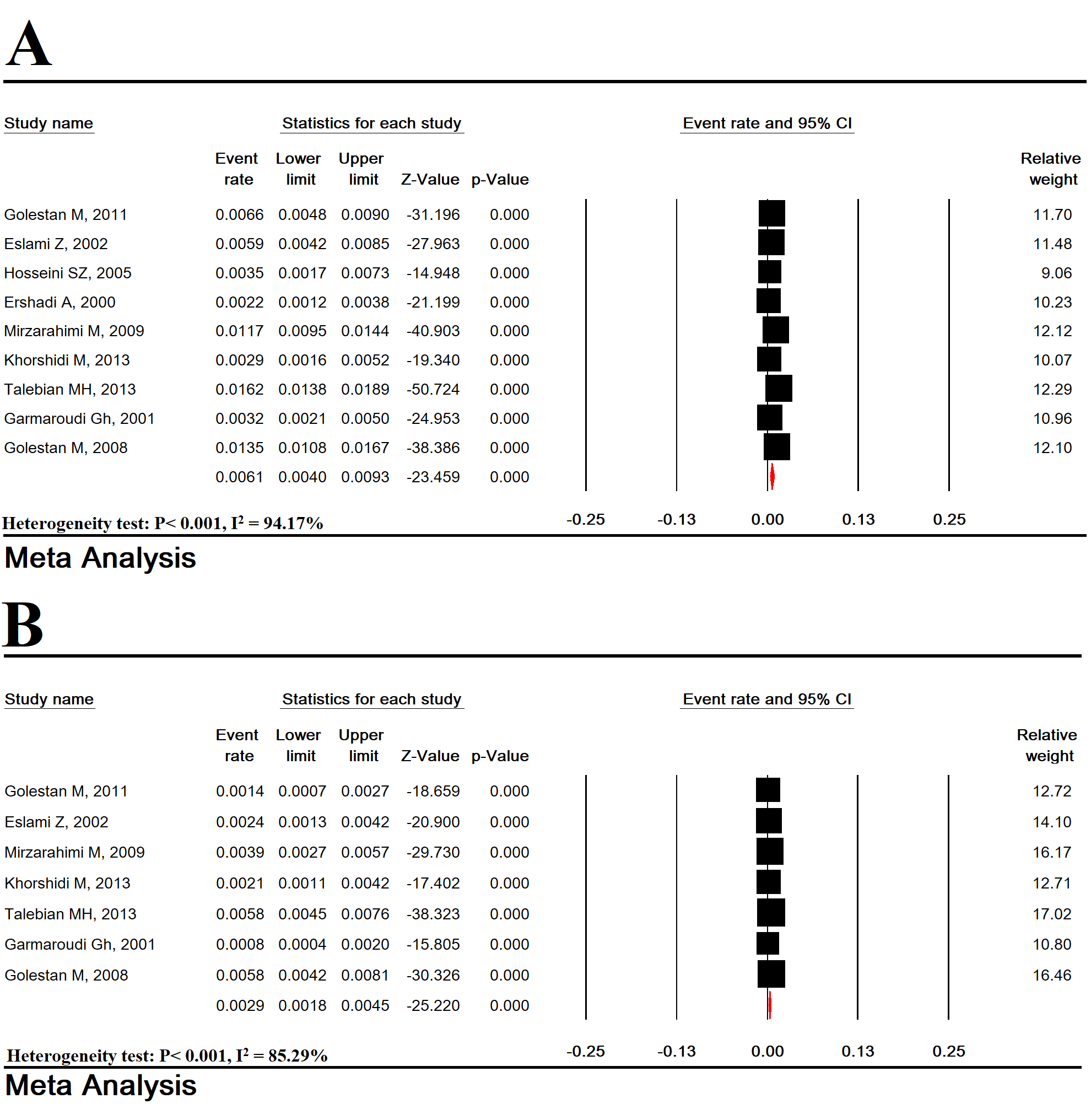
Figure 4: Sensitivity analysis for prevalence of VLBW (A), and ELBW (B)


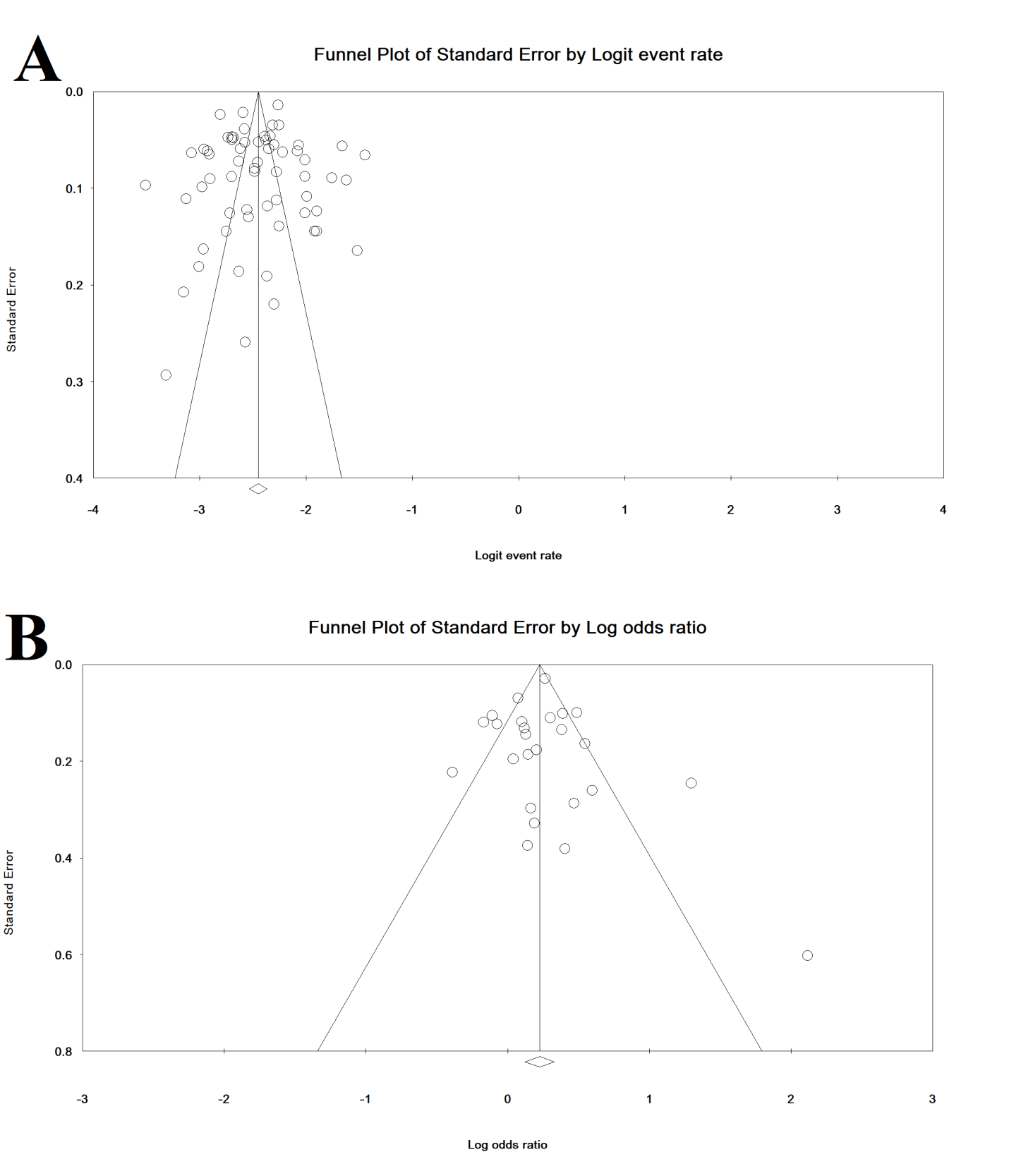
Figure 5: publication bias for studies of the prevalence of LBW (A) and an odds ratio of female to male (B)
